# R-loops Associated with Triplet Repeat Expansions Promote Gene Silencing in Friedreich Ataxia and Fragile X Syndrome

**DOI:** 10.1371/journal.pgen.1004318

**Published:** 2014-05-01

**Authors:** Matthias Groh, Michele M. P. Lufino, Richard Wade-Martins, Natalia Gromak

**Affiliations:** 1 Sir William Dunn School of Pathology, University of Oxford, Oxford, United Kingdom; 2 Department of Physiology, Anatomy and Genetics, University of Oxford, Oxford, United Kingdom; CABIMER, Universidad de Sevilla, Spain

## Abstract

Friedreich ataxia (FRDA) and Fragile X syndrome (FXS) are among 40 diseases associated with expansion of repeated sequences (TREDs). Although their molecular pathology is not well understood, formation of repressive chromatin and unusual DNA structures over repeat regions were proposed to play a role. Our study now shows that RNA/DNA hybrids (R-loops) form in patient cells on expanded repeats of endogenous *FXN* and *FMR1* genes, associated with FRDA and FXS. These transcription-dependent R-loops are stable, co-localise with repressive H3K9me2 chromatin mark and impede RNA Polymerase II transcription in patient cells. We investigated the interplay between repressive chromatin marks and R-loops on the *FXN* gene. We show that decrease in repressive H3K9me2 chromatin mark has no effect on R-loop levels. Importantly, increasing R-loop levels by treatment with DNA topoisomerase inhibitor camptothecin leads to up-regulation of repressive chromatin marks, resulting in *FXN* transcriptional silencing. This provides a direct molecular link between R-loops and the pathology of TREDs, suggesting that R-loops act as an initial trigger to promote *FXN* and *FMR1* silencing. Thus R-loops represent a common feature of nucleotide expansion disorders and provide a new target for therapeutic interventions.

## Author Summary

Friedreich ataxia and Fragile X syndrome are among 40 human diseases associated with expansion of repeated sequences. In both disorders repeat expansion leads to gene silencing, the molecular mechanism of which is not well understood, impeding the development of specific therapies to treat these disorders. It is proposed that formation of unusual DNA structures (RNA/DNA hybrids, or R-loops) over repeat regions may play a role, but their molecular function has not been investigated *in vivo*. We show that R-loops form on expanded repeats of *FXN* and *FMR1* genes in cells from FRDA and FXS patients. These R-loops are stable, correlate with repressive chromatin marks and hinder *FXN* transcription in patient cells. We studied the relationship between repressive chromatin and R-loops. Decrease in the amount of repressive chromatin has no effect on R-loop levels. In contrast, increase in the R-loops leads to transcriptional silencing of *FXN* gene and formation of repressive chromatin, providing a direct molecular link between R-loops and pathology of expansion diseases. This discovery is important for understanding the basic molecular mechanism underlying the pathology of expansion diseases. The ability of R-loops to trigger transcriptional silencing makes them an attractive target for future therapeutic approaches to treat these diseases.

## Introduction

Around forty human diseases are associated with expanded repeat sequences [1]. Friedreich ataxia (FRDA) is the most frequent autosomal recessive ataxia (2–4 cases/100,000), caused by a GAA expansion in the first intron of the frataxin (*FXN*) gene, which encodes a mitochondrial protein involved in iron-sulfur cluster biogenesis [2], [3]. The GAA expansion leads to reduced levels of *FXN* mRNA and protein [4]–[6]. Several mechanisms mediating *FXN* transcriptional silencing have been proposed, including the formation of unusual DNA structures (triplex DNA and RNA/DNA hybrids) and repressive heterochromatin over expanded repeats [5]–[10].

RNA/DNA hybrids (R-loops) are formed during transcription, when nascent RNA hybridizes to the DNA template behind the elongating RNA polymerase (Pol II). R-loops are detected in organisms from bacteria to humans and implicated in many processes [11]. In mammalian cells, R-loops were originally discovered in the immuno-globulin class switch regions, essential for generating the antibody diversity in mouse activated B cells [12], [13]. R-loops also accumulate in cells depleted of the key splicing factor SRSF1, resulting in genome instability and appearance of double-strand breaks [14]. Recent studies demonstrated that R-loops are enriched over CpG promoters and may be involved in protection of these regions from DNA methylation and maintaining the hypomethylated state of CpG promoters [15]. We recently showed that R-loops formed over the G-rich pause sites downstream of the polyA signal in human genes are essential for the process of transcriptional termination of RNA Pol II [16]. Furthermore RNA/DNA hybrids are induced at GAA repeats following *in vitro* transcription and in bacteria [17], [18]. Also R-loops formed on plasmids containing CTG/CAG repeats in *E.coli* and mini-gene constructs in human cells promoted repeat instability, pointing towards their role in disease pathology [19], [20]. However, the direct involvement of R-loops on endogenous expanded alleles in the pathology of FRDA has not yet been investigated *in vivo*.

Our study shows that RNA/DNA hybrids (R-loops) form on expanded repeats of endogenous *FXN* and *FMR1* genes, associated with Friedreich ataxia and Fragile X (FXS) disorders, in patient cells. These transcription-dependent R-loops are resistant to cellular degradation and co-localise with repressive H3K9me2 chromatin marks, characteristic of these diseases. Using nascent nuclear run-on analysis we show that R-loops over expanded repeats impede RNA Polymerase II transcription of the *FXN* gene in patient cells. We investigated the interplay between repressive chromatin marks and R-loops on the *FXN* gene. We show that a decrease in repressive H3K9me2 chromatin mark has no effect on R-loop levels and *FXN* transcription. In contrast, increasing R-loop levels leads to transcriptional repression of *FXN* gene, providing a direct molecular link between R-loops and pathology of FRDA. These data suggest that R-loops formed over expanded repeats act as an initial trigger to promote *FXN* and *FMR1* silencing, and represent a common feature of nucleotide expansion diseases, contributing to their pathology *in vivo*.

## Results

### *FXN* transcriptional initiation and elongation defect in FRDA cells

**Figure 1:**
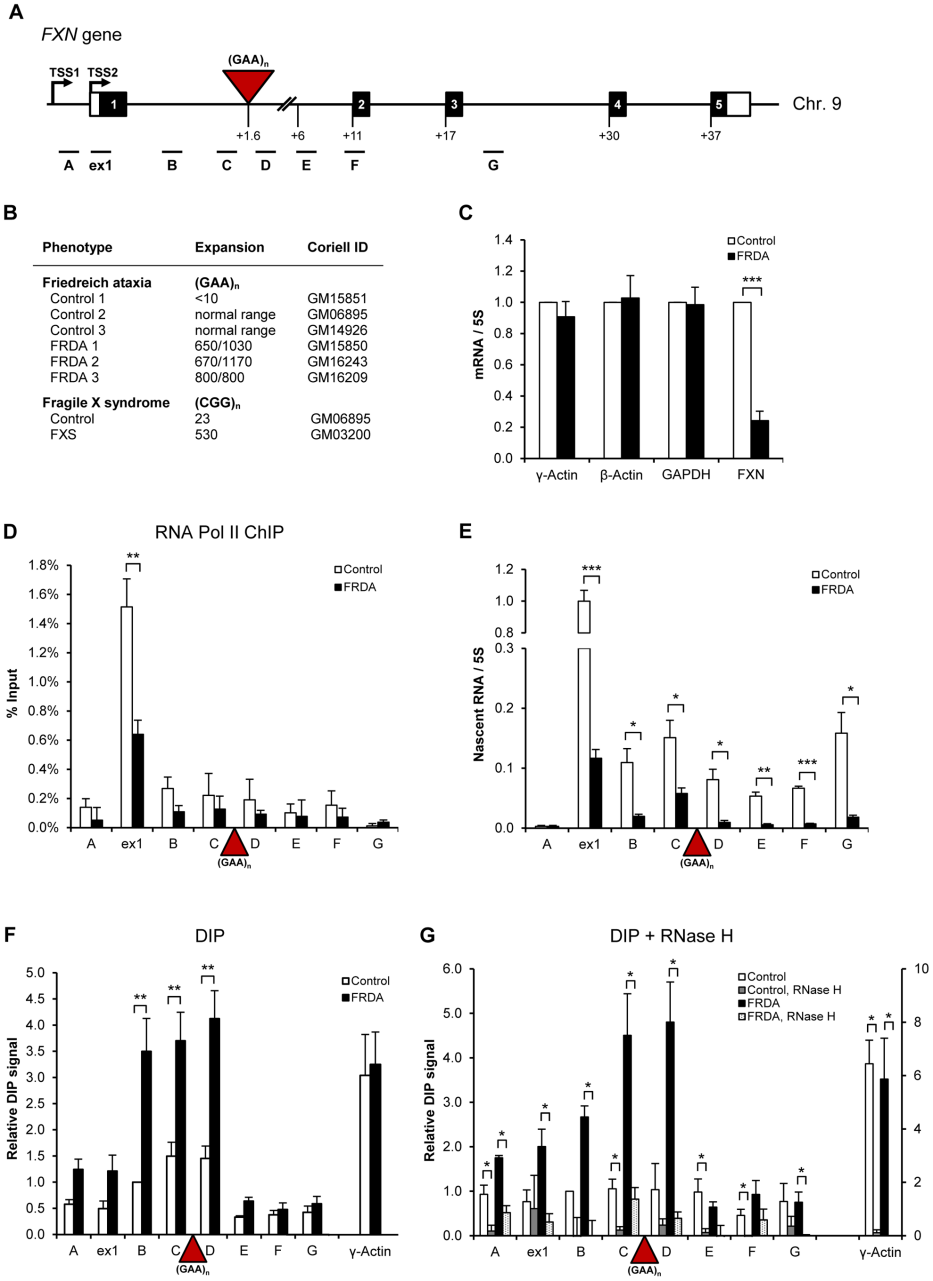
R-loops are formed over expanded repeats of *FXN* gene in FRDA cells. A. Diagram of *FXN* gene. Black boxes are exons, white boxes are 5′ and 3′UTRs, lines are introns, red triangle is (GAA)_n_ expansion. TSS2 is the major transcriptional start site in lymphoblastoid cells. qPCR amplicons are shown below the diagram. Numbers indicate the distances to TSS2 in kilobases. B. Cell lines used in the study. The repeat sizes are indicated. C. RT-qPCR analysis of γ-actin, β-actin, GAPDH and *FXN* mRNAs in control (GM15851) and FRDA (GM15850) cells. Values are normalised to 5S rRNA and relative to control cells. D. RNA Pol II ChIP in control (GM15851) and FRDA (GM15850) cells. E. RT-qPCR analysis of *FXN* nascent RNA in control (GM15851) and FRDA (GM15850) cells, normalised to 5S rRNA and relative to ex1 RNA in control cells. F. DIP on endogenous *FXN* gene in control (GM15851) and FRDA (GM15851) cells. γ-actin is positive control. G. R-loops are sensitive to RNase H digestion. DIP samples were treated with 25 U of recombinant *E.coli* RNase H (NEB, M0297S) for 6 hours at 37°C. γ-actin is positive control. Bars in C–G are average values +/− SEM (n>3).

We examined transcriptional regulation of the *FXN* gene in immortalized lymphoblastoid cells derived from FRDA patients, where *FXN* mRNA expression is reduced by ∼80% (Figure 1A–C). Pol II chromatin immuno-precipitation (ChIP) analysis in these cells showed that Pol II is enriched over the exon 1, positioned at the major transcriptional start site (TSS2) in lymphoblasts, correlating with the promoter-specific histone H3 depleted region [4] (Figure 1D, S1). Pol II levels over exon 1 were significantly reduced in FRDA cells. Similarly, using quantitative RT-PCR (RT-qPCR) in three independent control and three FRDA cell lines, a dramatic reduction in nascent RNA was detected over exon 1 in FRDA cells, further confirming a defect in transcription initiation (Figure 1E, S2A and S2B left panels). We also observed ∼10-fold reduction in the nascent RNA downstream of the expansion in regions D–G in FRDA cells. Overall Pol II ChIP and RT-qPCR results suggest transcriptional initiation and elongation defects triggered by expanded repeats, in line with previous reports [4], [6], [21].

### R-loops are formed over expanded repeat regions of *FXN* gene *in vivo*

Recently we established the DNA immuno-precipitation (DIP) method, which allows detection of R-loops on endogenous human genes *in vivo* using S9.6 antibody which recognizes RNA/DNA hybrids [16], [22]. Here we employed DIP to investigate R-loop distribution on the *FXN* gene. As a positive control we used the intron 1 region of the γ-actin gene, where high levels of R-loops are detected [15]. Significantly, we observed ∼3-fold enrichment of R-loops over regions B, C and D in the *FXN* intron 1 in FRDA cells, compared to control cells (Figure 1F). R-loops were concentrated over the expanded repeat region and were low in the downstream regions E–G. γ-actin R-loop levels were similar in control and FRDA cells (Figure 1F). Similar R-loop enrichment over expanded GAA repeats was detected in two additional independent FRDA cell lines (Figure S2A–B, right panels). Interestingly, when we compared the DIP data from all control and FRDA cell lines we observed that the level of R-loops correlates with expansion length (Figure S2C). To confirm the specificity of the DIP signal, we treated the samples with RNase H, which specifically degrades the RNA in RNA/DNA hybrids, prior to immuno-precipitation. Following RNase H digestion, the signal was strongly reduced for control γ-actin and *FXN* regions, suggesting that genuine R-loops are formed over the expanded GAA repeats (Figure 1G). High level of R-loops detected in FRDA cells may also suggest that these structures are particularly stable over *FXN* expanded repeats. Therefore, R-loops could act in *cis* to affect *FXN* gene expression in FRDA cells.

### R-loops are stable and impede Pol II transcription on *FXN* gene

**Figure 2:**
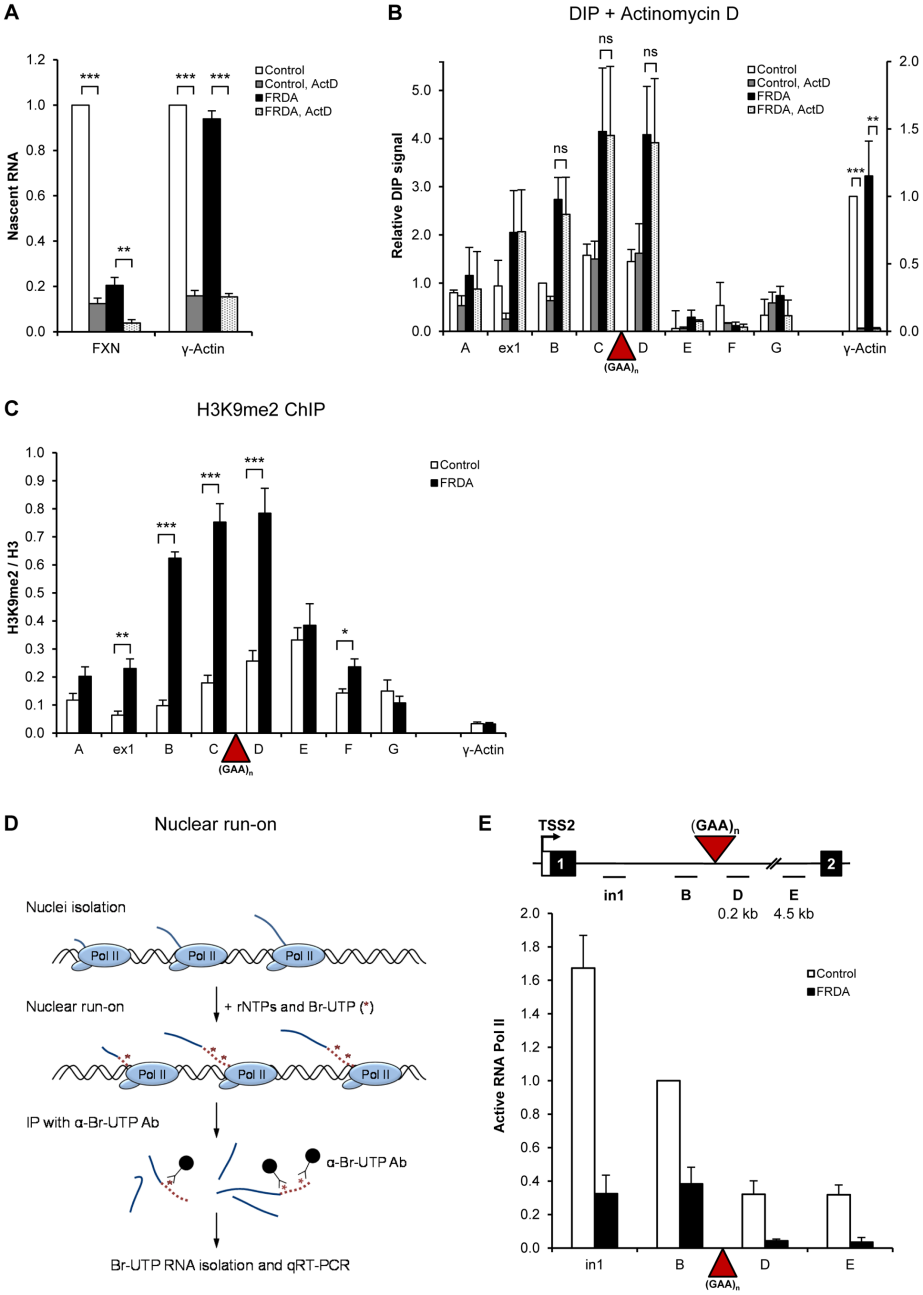
R-loops are stable and impede Pol II transcription on *FXN* gene. A. RT-qPCR analysis of nascent γ-actin and *FXN* RNA from control and FRDA cells treated with 5 µg/ml of actinomycin D for 21 hours. Values are relative to untreated control cells. B. DIP on *FXN* gene in control and FRDA cells treated with 5 µg/ml of actinomycin D for 21 hours. γ-actin is positive control. C. H3K9me2 ChIP in control and FRDA cells. H3K9me2 levels were normalized to the total H3 levels. γ-actin is used as background control. D. Diagram depicting the Br-UTP nuclear run-on (NRO) method. E. Br-UTP nuclear run-on in two control (GM15851, GM14926) and two FRDA (GM15850 and GM16243) cells, normalised to the region B in control cells. Bars in A–C and E are average values +/− SEM (n>3).

To understand the function of R-loops in FRDA pathology, we further characterized these structures over the expanded *FXN* allele. In particular, we treated cells with the transcriptional inhibitor actinomycin D. Following this treatment for 21 hours we observed an ∼80% reduction in γ-actin nascent RNA and R-loop signal, suggesting that γ-actin R-loops are quickly turned over in the cell (Figure 2A, B). In contrast, although nascent *FXN* RNA decreased following actinomycin D treatment, no change in R-loop levels was detected. However, we did finally observe a significant decrease in the level of R-loops over expanded repeats following prolonged treatment with actinomycin D for 48 hours (Figure S3). Overall these results suggest that R-loops associated with *FXN* expanded repeats are resistant to degradation. This may relate to the expanded GAA repeat property of transcriptional repression and repeat instability. We also observed that enrichment of R-loops correlated with highest peaks of the repressive histone modification H3K9me2 over the *FXN* regions B–D in FRDA cells (Figure 2C and 1F). This suggests that R-loops over expanded GAA repeats may functionally associate with repressive heterochromatin and be involved in mediating transcriptional repression.

Previously we showed that R-loops formed at the 3′ends of human genes promote transcriptional termination of RNA Pol II [16]. We therefore investigated if R-loops over *FXN* expanded repeats affect Pol II elongation. Here we employed nuclear run-on (NRO) analysis with Br-UTP labelled nucleotide [16], which measures actively transcribing Pol II (Figure 2D), in contrast to ChIP, which detects the total Pol II level on the gene. Using NRO we observed a substantial decrease in active transcription upstream of the GAA expansion (regions in1 and B) in FRDA cells, confirming our Pol II ChIP results (Figure 2E and 1D). In addition, we also detected ∼3-fold reduction in active transcription in FRDA cells over *FXN* regions D and E, positioned 210 nt and 4.5 kb downstream of expansion, respectively (Figure 2E). This elongation defect is not due to the increased distance caused by GAA expansion (∼3 kb), since we observed no decrease in active transcription between regions D to E, separated by ∼4.3 kb, in both cell lines. This suggests that expanded repeats directly interfere with active Pol II transcription in FRDA cells.

### R-loops on expanded repeats of integrated *FXN* gene are sensitive to RNase H1 over-expression in HEK293 cells

**Figure 3:**
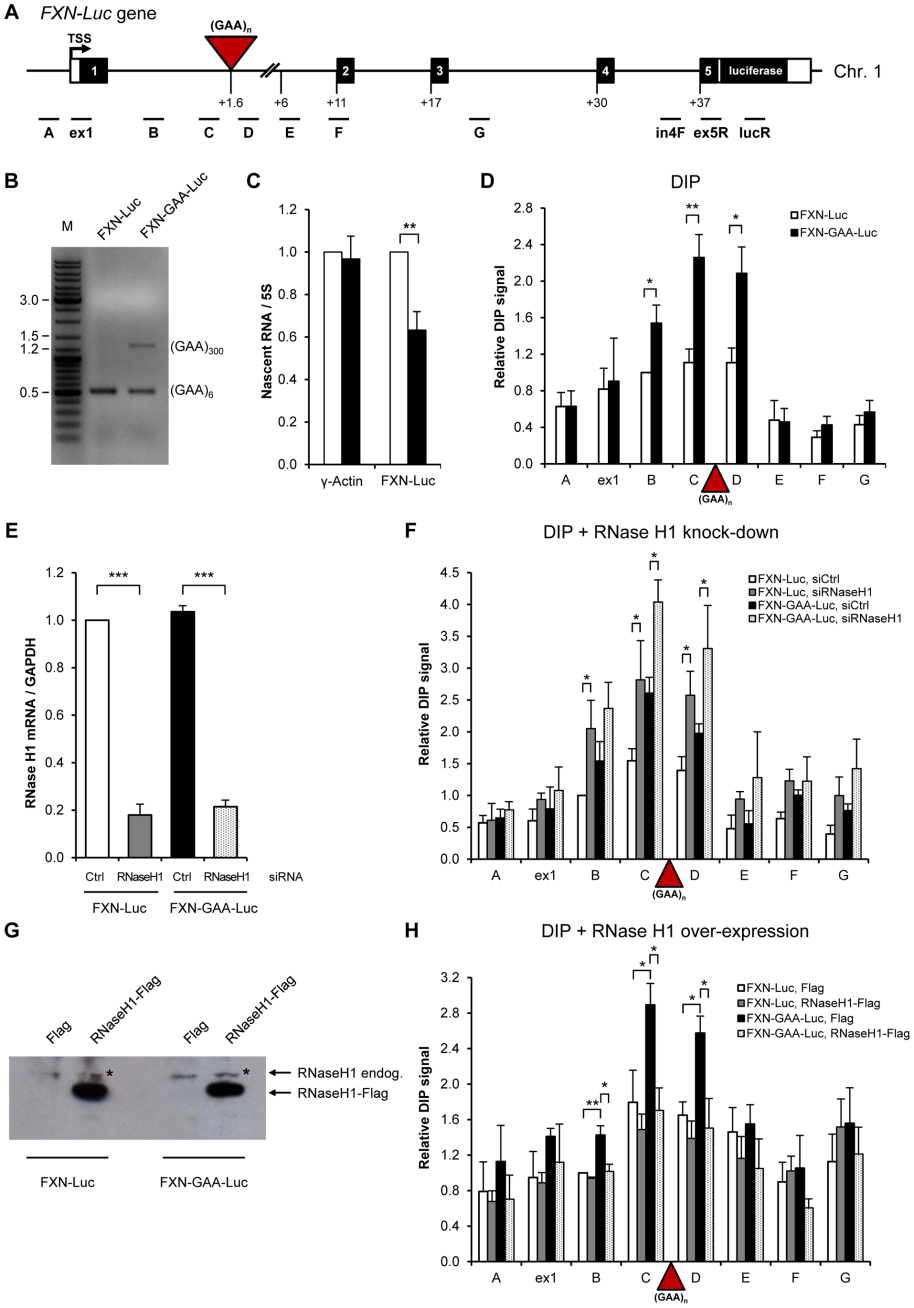
Over-expressed RNase H1 resolves R-loops formed on *FXN* expanded repeats in HEK293 cells. A. Diagram of the FXN-Luc gene, containing 6 (*FXN-Luc*) or 310 GAA repeats (*FXN-GAA-Luc*), integrated on the chromosome 1 of HEK293 cells. Frataxin gene was fused to the luciferase at the beginning of the *FXN* exon 5. Black boxes are exons, white boxes are 5′ and 3′UTRs, lines are introns, red triangle is (GAA)_n_ expansion. TSS is the transcriptional start site. qPCR amplicons are shown below the diagram. Numbers indicate the distances to TSS in kilobases. B. Size of GAA expansion determined by PCR analysis on genomic DNA from *FXN-Luc* and *FXN-GAA-Luc* cell lines, using GAA104F and GAA629R primers. PCR products were run on 1% agarose gel. M denotes the marker lane. *FXN-Luc* and *FXN-GAA-Luc* cells contain endogenous wild type *FXN* gene [23], giving rise to the PCR product of 0.5 kb. C. *FXN* and γ-actin nascent RNA levels in *FXN-Luc* (white bars) and *FXN-GAA-Luc* (black bars) HEK293 cells, determined by RT-qPCR and normalised to 5S rRNA. The level of *FXN* and γ-actin nascent RNA in *FXN-Luc* cells was taken as 1. LucR primer was used for the reverse transcription reaction. qPCR was carried using in4F and ex5R primers, shown in A. D. DIP analysis on *FXN-Luc* gene in *FXN-Luc* (white bars) and *FXN-GAA-Luc* (black bars) HEK293 cells using RNA/DNA hybrid-specific S9.6 antibody. E. RT-qPCR analysis of RNase H1 mRNA from *FXN-Luc* and *FXN-GAA-Luc* cells, treated with control and RNase H1 siRNAs. Values are normalised to GAPDH mRNA and are relative to *FXN-Luc* cells, treated with control siRNA. F. DIP analysis on *FXN-Luc* gene in *FXN-Luc* and *FXN-GAA-Luc* HEK293 cells, treated with control and RNase H1 siRNAs. G. Western blot analysis of 50 µg of protein extracts obtained from *FXN-Luc* and *FXN-GAA-Luc* cells transfected with Flag and RNase H1-Flag expression plasmids. Western blot was probed with anti-RNase H1 antibody. * denotes endogenous RNase H1 protein. H. DIP analysis on *FXN-Luc* gene in *FXN-Luc* and *FXN-GAA-Luc* HEK293 cells transfected with Flag or RNase H1-Flag expression plasmids. Bars in C–F and H represent the average values from at least three independent experiments +/− SEM.

To test if expanded GAA repeats trigger the formation of R-loops in a different genomic location, we employed HEK293 cells, containing a copy of the *FXN* gene with either six (*FXN-Luc*) or ∼310 (*FXN-GAA-Luc*) GAA repeats, fused to the luciferase gene, integrated on chromosome 1, while the endogenous *FXN* gene lies on chromosome 9 (Figure 3A) [23]. We confirmed the presence of the GAA expansion using PCR on genomic DNA extracted from these cells. As demonstrated in Figure 3B, the *FXN-GAA-Luc* cell line indeed contains ∼310 expanded repeats. The presence of GAA repeats caused a reduction of ∼37% in *FXN*-luciferase nascent RNA levels as determined by RT-qPCR (Figure 3C) and an increase in the repressive histone modification H3K9me2 (Figure S4A), recapitulating repression of gene expression seen in FRDA cells. This smaller reduction in the RNA levels in *FXN-GAA-Luc* cells compared to FRDA lymphoblastoid cells can be explained by low number of repeats (only ∼310) on the integrated *FXN* copy.

Next we investigated if R-loops are formed on expanded repeats of the integrated *FXN* copy in HEK293 cells using DIP analysis (Figure 3D). Similar to patient-derived FRDA lymphoblast cells, we observed 2–3-fold increase in the level of R-loops over expanded repeats region (amplicons B, C and D) in *FXN-GAA-Luc* cells. This suggests that R-loops are formed on transcribed expanded GAA repeats, independently of their genomic location. To confirm the specificity of this R-loop signal, we employed RNAi to knock down endogenous RNase H1 enzyme, which specifically degrades the RNA in RNA/DNA hybrids [24]. Following depletion of RNase H1 in HEK293 cells, we observed a significant increase in the R-loop signal, suggesting that endogenous RNase H1 can degrade R-loops formed over *FXN* gene *in vivo* (Figure 3E, F).

Next we wanted to test if RNase H1 over-expression can resolve R-loops formed on expanded GAA repeats. To this end we over-expressed Flag and RNase H1-Flag constructs in *FXN-Luc* and *FXN-GAA-Luc* cells. High level of RNase H1 over-expression was confirmed by RT-qPCR (Figure S4B) and western blot analysis (Figure 3G). Interestingly, following RNase H1 over-expression, we observed a reduction of R-loop signal over the GAA expansion (Figure 3H). In line with these observations, RNase H1 over-expression resulted in up-regulation of *FXN* transcription from the expanded allele (Figure S4C). This suggests that R-loops formed over expanded repeats *in vivo* can be resolved by over-expressed RNase H1 and removal of R-loops leads to increase in *FXN* gene expression.

### R-loops trigger *FXN* transcriptional repression *in vivo*

**Figure 4:**
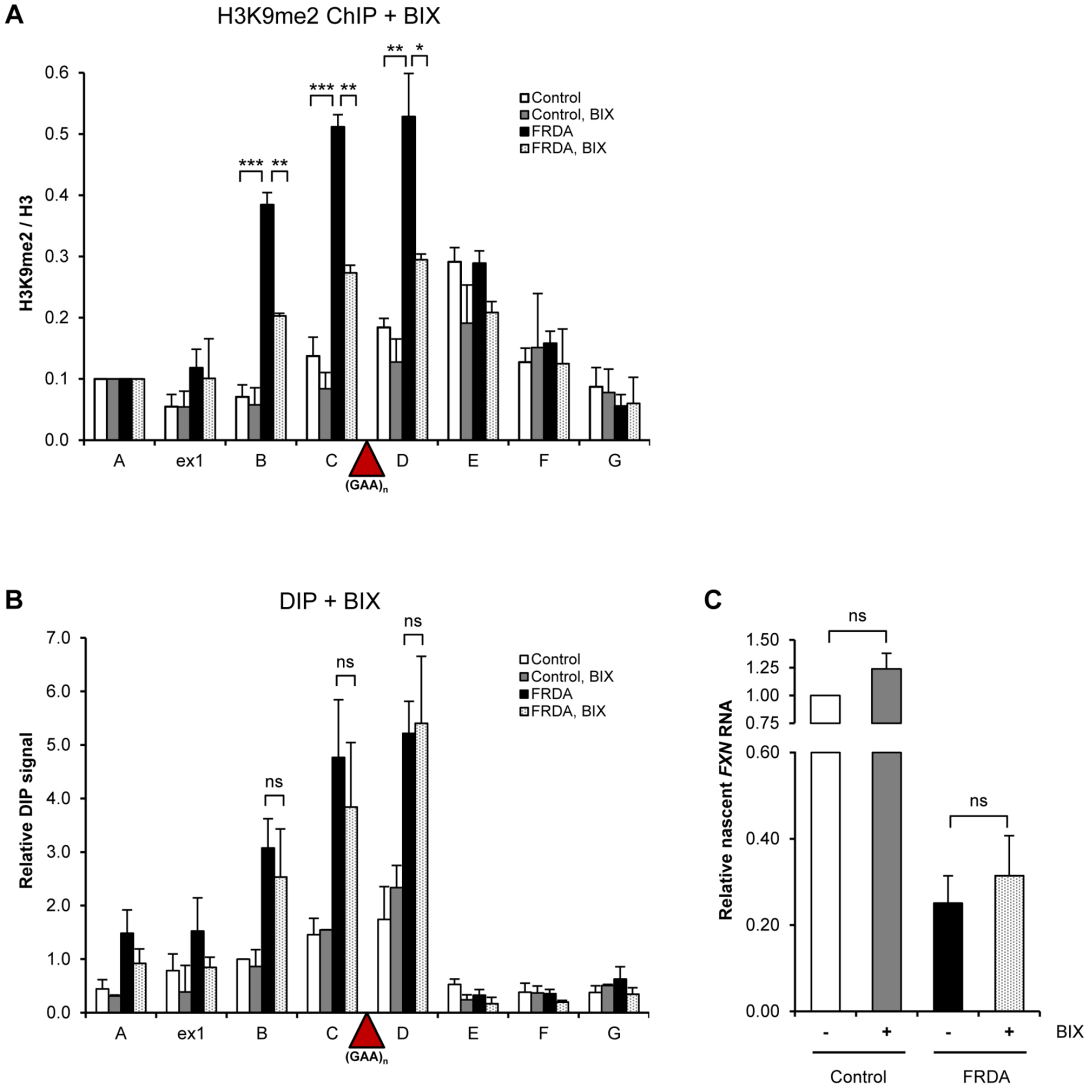
R-loops are not affected by changes in H3K9 dimethylation. A. H3K9me2 ChIP in control and FRDA cells, treated with 4 µM BIX-01294 for 72 h. H3K9me2 levels were normalized to the total H3 levels and relative to amplicon *FXN* A, not affected by the treatment. B. DIP analysis in control and FRDA cells, treated with 4 µM BIX-01294 for 72 h. C. RT-qPCR analysis of *FXN* nascent RNA in control and FRDA cells, treated with 4 µM BIX-01294 for 72 h. Values are relative to untreated control cells and normalized to γ-actin nascent RNA. Bars in A–C are average values +/− SEM (n>3).

Previous studies have demonstrated that expanded *FXN* GAA repeats are associated with increased levels of heterochromatin marks [4], [6], [7]. To investigate the relationship between R-loops and repressive heterochromatin marks formed on the expanded *FXN* allele, we employed histone methyltransferase inhibitor BIX-01294, previously shown to reduce the level of H3K9me2 over repeat regions [21]. Following BIX-01294 treatment, we observed a significant reduction in the levels of H3K9me2 chromatin mark (Figure 4A), similar to previous reports [21]. Significantly, following BIX-01294 treatment the level of R-loops over the expanded repeat region remained unchanged (Figure 4B). Similarly, reduction in H3K9me2 chromatin mark had no effect on *FXN* nascent RNA level (Figure 4C). These data suggest that H3K9me2 chromatin modification is not directly responsible for the *FXN* transcriptional repression and is likely to be a consequence of the reduced transcription or caused by R-loop formation.

**Figure 5:**
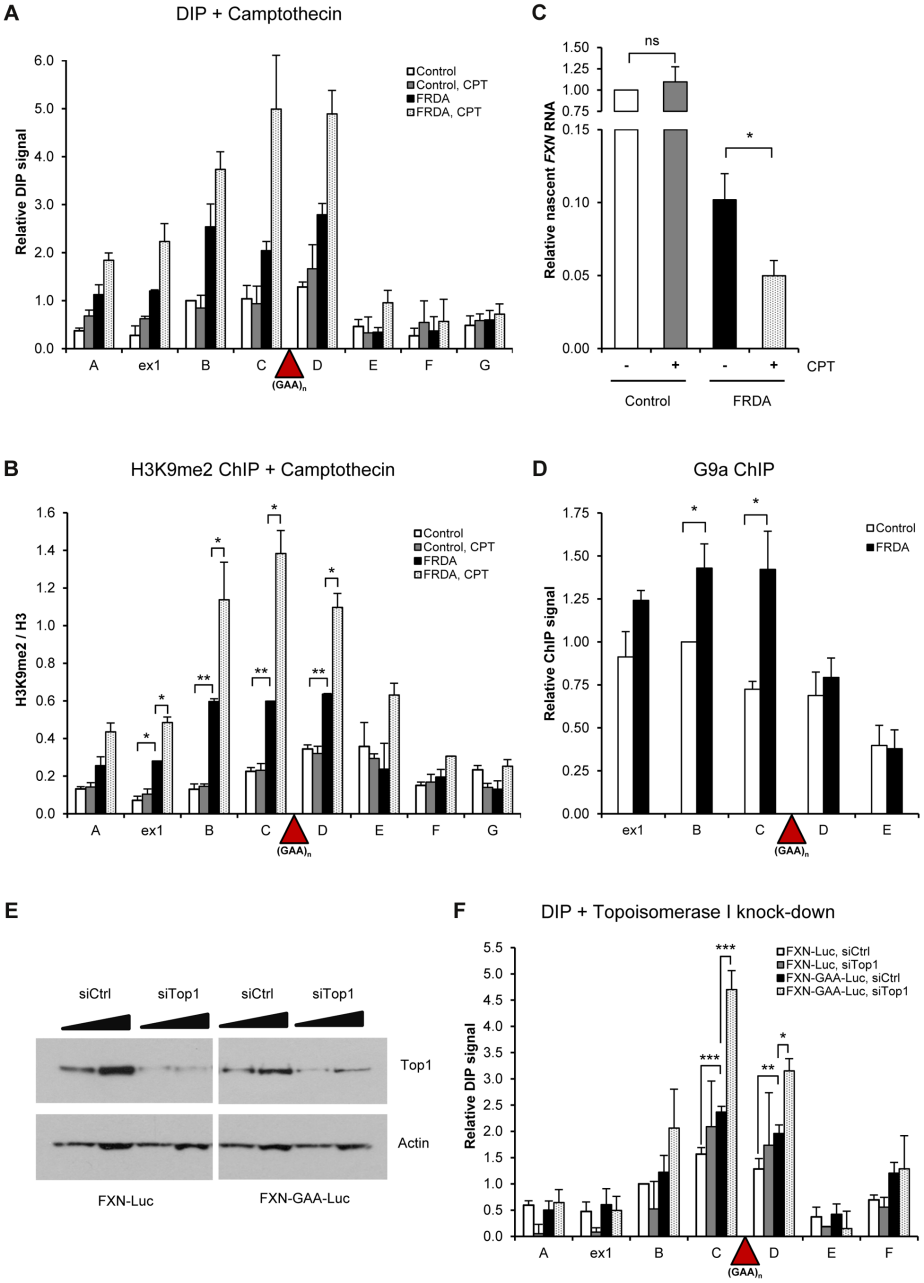
R-loops trigger transcriptional repression of *FXN* gene. A. DIP analysis on *FXN* gene in control and FRDA cells, treated with 10 µM camptothecin (CPT) for 6 hours. B. H3K9me2 ChIP on *FXN* gene in control and FRDA cells, treated with 10 µM camptothecin (CPT) for 6 hours. H3K9me2 levels were normalized to the total H3 levels. C. RT-qPCR analysis of *FXN* nascent RNA in control and FRDA cells, treated with 10 µM camptothecin for 6 hours. Values are relative to untreated control cells and normalized to γ-actin nascent RNA. D. G9a ChIP on *FXN* gene in control and FRDA cells. G9a levels are normalised relative to amplicon B in control cells. E. Western blot analysis of 20 and 40 µg of protein extracts obtained from *FXN-Luc* and *FXN-GAA-Luc* cells, treated with control and Top1 siRNAs. Western blot was probed with anti-Top1 and anti-actin antibody. F. DIP analysis on *FXN* gene in *FXN-Luc* and *FXN-GAA-Luc* HEK293 cells, treated with control and Top1 siRNAs. Bars in A–D and F are average values +/− SEM (n>3).

To investigate the ability of R-loops to directly trigger *FXN* transcriptional repression, we took advantage of camptothecin (CPT), a specific inhibitor of DNA Topoisomerase I (Top1), an enzyme which relieves transcription-induced DNA supercoiling. Loss of Top1 enhances R-loop formation in yeast and human cells [25], [26]. Following CPT treatment, we observed an increase in R-loop formation over the expanded repeat region in FRDA cells while R-loop levels in *FXN* regions E–G remained unchanged (Figure 5A). This effect was consistent between different patient-derived cell lines (Figure S5A). We detected no effect on R-loop levels in control cells, demonstrating the specificity of CPT treatment to expanded repeats (Figure 5A and S5A). The ability of CPT to increase R-loop levels was not due to CPT-induced covalent links between Top1 and DNA, since Top1 knock-down also resulted in R-loop accumulation (Figure 5E, F).

Interestingly, increase in the level of R-loops coincided with increase in the amount of repressive H3K9me2 mark over *FXN* regions B–D surrounding the expansion in FRDA cells (Figure 5B and S5B). This also resulted in down-regulation of nascent *FXN* RNA in FRDA cells, but not in control cells, as observed in three independent control and FRDA cell lines (Figure 5C and S5C–D). These data suggest that R-loops at expanded repeats directly trigger *FXN* transcriptional repression and promote formation of repressive H3K9me2 marks. To understand the molecular mechanism of this process, we investigated the binding of G9a histone methyltransferase, which deposits H3K9me2 marks on histones, to *FXN* gene. Interestingly, we observed that G9a is enriched over the expanded region in FRDA cells (Figure 5D). This suggests that R-loops may recruit G9a to the expanded repeat regions, thereby promoting the formation of repressive H3K9me2 marks.

### R-loops are formed over (CGG)_n_ expanded repeats of *FMR1* gene

**Figure 6:**
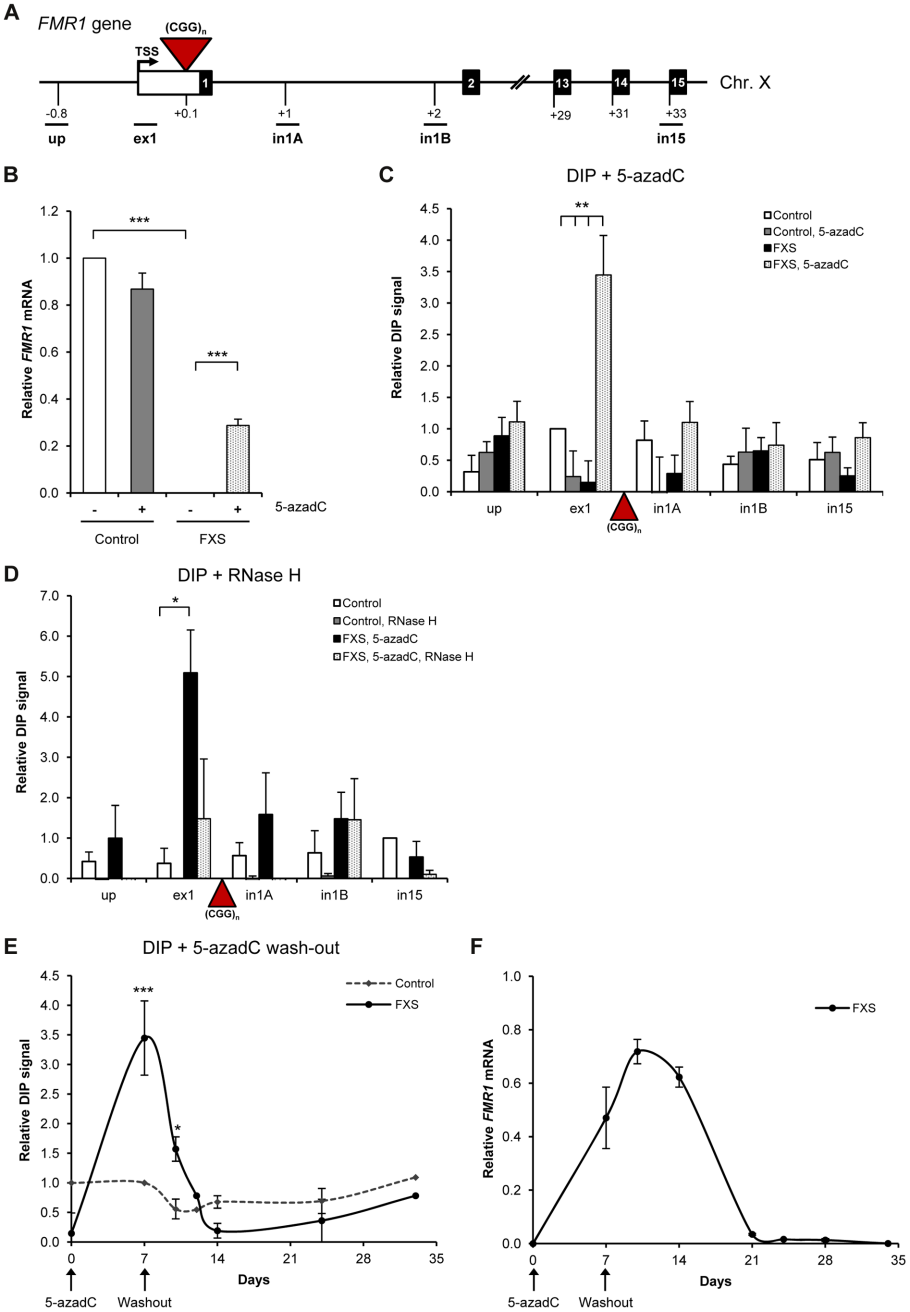
R-loops are formed over (CGG)_n_ expanded repeats of *FMR1* gene. A. Diagram of *FMR1* gene. Black boxes are exons, white box is 5′ UTR and lines are introns. Red triangle is (CGG)_n_ expansion. qPCR amplicons are shown below the diagram. TSS is the transcriptional start site. Numbers indicate the distances to TSS in kilobases. B. RT-qPCR analysis of *FMR1* mRNA in control and FXS cells, treated with 1 µM 5-azadC for 7 days, normalized to GAPDH. C. DIP analysis on endogenous *FMR1* gene in control and FXS cells, treated with 1 µM 5-azadC for 7 days. Values are relative to ex1 region in control untreated cells. D. *FMR1* R-loops are sensitive to RNase H digestion, following the treatment with 25 U of RNase H for 6 hours at 37°C prior to immuno-precipitation. Values are relative to in15 region in control untreated cells. E. R-loop kinetics on exon 1 of *FMR1* gene in control and FXS cells during the process of transcriptional re-activation with 1 µM 5-azadC (7 days) followed by 5-azadC wash out with drug-free media (28 days). Values are relative to ex1 region in control untreated cells on day 7. F. RT-qPCR analysis of *FMR1* mRNA in control and FXS cells, treated with 1 µM 5-azadC (7 days) followed by 5-azadC wash out with drug-free media (28 days). The level of *FMR1* mRNA in control cells is taken as 1. Bars in B–D are average values +/− SEM (n>3).

To test if R-loop formation is a general feature of trinucleotide expansion diseases, we also examined the *FMR1* gene. In Fragile X syndrome patients, the *FMR1* allele containing a (CGG)_n>200_ expansion in the 5′UTR is fully methylated and transcriptionally silenced [27]. Therefore to investigate the potential role of R-loops in FXS, *FMR1* transcription was reactivated by treatment with the DNA methylation inhibitor 5-aza-2′-deoxycytidine (5-azadC). This resulted in expression of *FMR1* mRNA in FXS cells to 25% of control cells, as previously reported [28]. However, *FMR1* expression was unchanged in control cells (Figure 6B). Using DIP, we detected low R-loop signal in control and untreated FXS cells (Figure 6C). Following 5-azadC treatment, we observed ∼4-fold increase in R-loops over the exon 1 region upstream of the expansion in FXS cells, while no significant changes were detected in control cells. The specificity of the DIP signal was confirmed by RNase H treatment (Figure 6D). These data suggest that R-loops are transcriptionally-dependent and localise to the expanded (CGG) repeat region. Since inhibition of DNA methylation only partially reactivates expanded *FMR1* allele in FXS cells, it is possible that R-loops at expanded (CGG) repeats prevent full reactivation.

To further characterize the relationship between R-loops and *FMR1* expression, we carried out kinetic experiments. In particular, we studied R-loop and *FMR1* mRNA levels during the process of transcriptional re-activation with 5-azadC treatment (7 days) followed by 5-azadC wash out with drug-free media for 28 days (Figure 6E, F). We observed that the R-loop levels over the exon 1 of *FMR1* gene stayed at the background during activation and wash-out period in control cells (dotted line in Figure 6E). In FXS cells, the R-loops were at their peak during the re-activation procedure with 5-azadC on day 7. After removal of 5-azadC, R-loop levels gradually diminished and completely disappeared after 7 days (day 14 of the full experiment). This pattern of R-loop dynamics correlated with *FMR1* expression profile (Figure 6F), suggesting that R-loops are associated with *FMR1* gene regulation.

## Discussion

We demonstrate that R-loops are formed over endogenous expanded (GAA) and (CGG) repeats *in vivo*, associated with FRDA and FXS disorders, respectively (Figure 1, 6). We show that these R-loops interfere with nascent Pol II transcription on *FXN* gene (Figure 2E). We also demonstrate that R-loops can trigger gene silencing irrespectively of their genomic location (Figure 3). R-loops over expanded repeats are very stable in human cells (Figure 2B), possibly due to failure of their complete turn-over by endogenous enzymes, which may contribute to FRDA pathology. Interestingly, expansion-associated R-loops can be resolved by over-expressed exogenous RNase H1, which leads to transcription up-regulation of *FXN* expression *in vivo* (Figure 3).

Previous work has demonstrated that co-transcriptionally formed RNA/DNA hybrids mediate transcription elongation impairment *in vitro* and in yeast *S.cerevisiae* [29], [30], suggesting that R-loops may provide roadblocks for RNA polymerases. R-loops over expanded repeats may form a structural block, directly interfering with Pol II transcription elongation. Similar to R-loops at the 3′ends of human genes [16], expansion-associated R-loops could promote RNA Pol II termination, resulting in reduction of active Pol II downstream of the expansion, as detected in this study.

**Figure 7:**
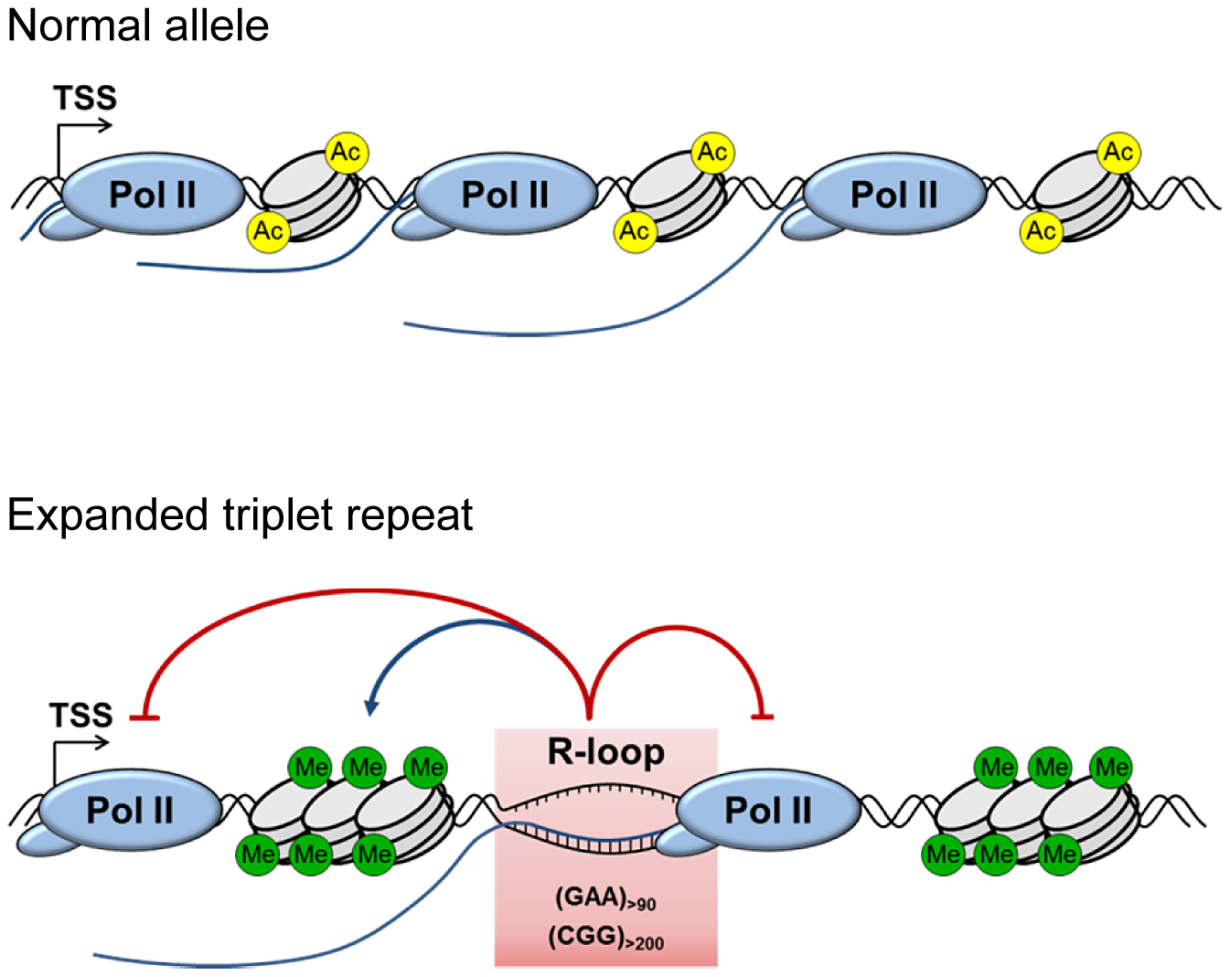
Model for the role of R-loops in mediating *FXN* and *FMR1* gene silencing. Background R-loop level on wild type allele allows efficient transcriptional elongation and gene expression. Transcribed (GAA)_n_ and (CGG)_n_ expanded repeats form R-loops resulting in decreased initiation and elongation of RNA Pol II. This leads to downregulation of *FXN* and *FMR1* expression, associated with formation of repressive DNA and chromatin marks.

Recently it was suggested that repressive chromatin H3K9me2 modification was not directly responsible for the *FXN* transcriptional repression [21]. In line with this, reversal of repressive DNA methylation on *FMR1* gene was not sufficient to fully restore *FMR1* expression [28]. We now show that a decrease in the level of the repressive H3K9me2 chromatin mark does not result in decrease of R-loops on the expanded allele or up-regulation of *FXN* RNA (Figure 4). These data indicate that R-loop formation is an early event in the process of *FXN* transcriptional gene silencing, which happens prior to the appearance of heterochromatin marks. We also show that increasing R-loop levels lead to an increase in repressive chromatin marks and subsequent repression of *FXN* gene expression (Figure 5). Furthermore, we observed that recruitment of G9a methyltransferase is enhanced on expanded *FXN* allele (Figure 5), providing an interesting possibility that R-loops may directly recruit this enzyme to promote H3K9me2 histone mark deposition. Altogether our results suggest that R-loops act as the primary trigger for repression of expanded *FXN* and *FMR1* alleles which may in turn act to promote heterochromatin formation. Consistent with our data, recently it has been demonstrated that promoter-bound trinucleotide repeat-containing mRNA induces epigenetic silencing in Fragile X syndrome [31]. Indeed R-loops have been implicated in the formation and maintenance of heterochromatin at centromeres in *S. pombe* [32]. In line with this, H3K9me2 modification is also enriched at the R-loop-containing pause region of β-actin gene (Figure S6), essential for Pol II transcriptional termination [16]. This suggests that R-loops may promote H3K9me2 modification at the 3′end of this gene, similar to expanded repeats of *FXN* gene. We propose that R-loops may be a common feature of many trinucleotide expansion diseases, contributing to their pathology *in vivo* (Figure 7). The ability of R-loops to trigger transcriptional silencing in trinucleotide expansion diseases makes them an attractive target for future therapeutic approaches to treat these devastating diseases.

In addition to uncovering the molecular mechanisms underlying FRDA and Fragile X pathologies, our work also provides interesting implications for R-loop biology. Taken into consideration this work and the work of others in the field, depending on their genomic location, R-loops may have different functions (reviewed in [11]). Therefore, stable R-loops formed over expanded triplet repeats (this study) may be different from R-loops at the 3′-ends of genes [16], [33] and R-loops formed over CpG island promoters [15], [33]. At promoters, R-loops play a protective role against epigenetic silencing. By contrast, R-loops over *FXN* expanded repeats correlate with a reduction in transcription elongation and the enrichment of repressive chromatin marks. This suggests that R-loops may be ‘sensed’ differently, depending on their genomic location and sequence composition. Understanding the molecular mechanisms that cells use to distinguish ‘harmful’ from ‘useful’ R-loops is an important biological question in the study of human diseases.

## Materials and Methods

### Cell culture and drug treatments

Epstein-Barr virus (EBV)-transformed lymphoblastoid cell lines from healthy (GM15851, GM14926, GM06895), FRDA (GM15850, GM16243, GM16209) and FXS (GM03200) patients were obtained from Coriell Institute for Medical Research. Frataxin (GAA) repeat sizes were 650/1030 (GM15850), 800/800 (GM16209) and 670/1170 (GM16243). (CGG) repeat size in *FMR1* gene was 530 (GM03200). All experiments were performed with early passages of the cell lines. Lymphoblastoid cell lines were grown in RPMI 1640 medium supplemented with 15% fetal bovine serum (FBS), 100 U/ml penicillin and 100 µg/ml streptomycin at 37°C in 5% CO_2_. 1 µM 5-azadC (Sigma, A3656) was added to the media for 7 days. 4 µM BIX-01294 (Sigma, B9311) was added to the media for the total of 72 hours. The media was replenished every 24 hours by replacement of half of the conditioned media with fresh media and drug. 10 µM camptothecin (Sigma, C9911) was added for 6 hours. Actinomycin D (Sigma, A9415) was added to a final concentration of 5 µg/ml to the media for 6–48 hours. *FXN-Luc* and *FXN-GAA-Luc* HEK293 cells were described in [23] and cultured in DMEM medium supplemented with 10% FBS, 100 U/ml penicillin/streptomycin, 100 µg/ml hygromycin B (Life Technologies). For 5-azadC wash-out experiments, cells were treated with 1 µM 5-azadC for 7 days. On day 7, cell were washed twice with fresh RPMI 1640 medium and cultured in the absence of 5-azadC during the indicated time.

### ChIP analysis

ChIP analysis on endogenous genes was carried out as previously described [34], [35]. 5 µg of the following antibodies were used: Pol II antibody (Santa Cruz, H-224), H3 (AbCam, ab1791), H3K9me2 (AbCam, ab1220), G9a (AbCam, ab40542). The immuno-precipitated DNA was used as template for real-time quantitative PCR performed using a Rotor-Gene RG-3000 machine (Corbett Research). The PCR mixture contained QuantiTect SYBR green PCR master mix (Qiagen), 2 µl of the template DNA and corresponding primers from Table S1. Cycling parameters were 95°C for 15 min, followed by 45 cycles of 94°C for 20 s, 58°–62°C for 20 s, and 72°C for 20 s. Fluorescence intensities were plotted against the number of cycles by using an algorithm provided by the manufacturer. Amount of immuno-precipitated protein at a particular gene region was calculated as ‘% of Input’ after subtracting the background signal, as determined by the ‘no antibody’ control.

### Expansion PCR in HEK293 cells

20 ng of genomic DNA was used as template in a 25 µl PCR reaction, containing 2.5 U Biotaq DNA polymerase, 3 mM MgCl_2_, 0.4 mM dNTPs, 0.4 µM GAA104F primer, 0.4 µM GAA629R primer in 1× NH4 reaction buffer. Products were amplified using protocol from [36] with minor modifications. In particular, 5 min at 94°C were followed by 10 cycles of 94°C for 20 s, 65°C for 30 s, 72°C for 5 min. This was followed by 20 cycles of 94°C for 20 s, 65°C for 30 s and 72°C for 5 min, with the 72°C step becoming 20 s longer in each cycle. After a final step at 72°C for 10 min, PCR products were resolved on a 1% agarose gel.

### DIP analysis

DNA immuno-precipitation (DIP) analysis on endogenous genes was performed with antibody, recognising RNA/DNA hybrids, purified from S9.6 hybridoma cell lines [37], as described in [16]. In particular, lymphoblastoid and HEK293 cells were split one day before DIP. 10×10^6^ cells were harvested, washed in PBS and incubated in cell lysis buffer (85 mM KCl, 5 mM PIPES pH 8.0, 0.5% NP-40) for 10 min on ice. Nuclei were collected by centrifugation and then incubated in nuclei lysis buffer (50 mM TRIS pH 8.0, 5 mM EDTA, 1% SDS) on ice. Proteins were digested by incubation with proteinase K (Roche) for 3 h at 55°C. Proteins and cell debris were removed by centrifugation after addition of KOAc to the final concentration of 1 M. Genomic DNA containing R-loops was then precipitated by addition of isopropanol. After washing the DNA pellet with 70% EtOH, genomic DNA was resuspended in 400 µl IP dilution buffer (16.7 mM TRIS pH 8.0, 1.2 mM EDTA, 167 mM NaCl, 0.01% SDS, 1.1% Triton X-100) and used for sonication (Diagenode Bioruptor). Bioruptor (Diagenode) settings were 3 min sonication, at the Medium setting 30 sec on/30 sec off interval and the average size of the fragments was ∼500 nt. Sonicated genomic DNA was then pre-cleared with 50 µl protein A agarose beads (Millipore) in 3 ml IP dilution buffer including protease inhibitors (0.5 mM PMSF, 0.8 µg/ml pepstatin A, 1 µg/ml leupeptin) for 1 h at 4°C. 10 µl of S9.6 antibody was added to DNA corresponding to 10×10^6^ cells. Immuno-precipitation was carried out over night at 4°C. Subsequent washes and elution steps are identical to the procedure as described for ChIP.

The immuno-precipitated, non-precipitated, and input DNAs were used as templates for qPCR. DIP RNase H-sensitivity analysis was carried out following the genomic isolation and prior to immuno-precipitation step with the addition of 25 U of RNase H (NEB, M0297S). 100 µl nuclease digestion reaction contained 1× reaction buffer, and it was performed for 6 hours at 37°C. Amount of immuno-precipitated RNA/DNA hybrid at a particular gene region was calculated as ‘% of Input’ after subtracting the background signal, as determined by the ‘no antibody’ control. In the case of *FXN* gene, all the values are relative to the *FXN* B amplicon in control cells.

### RNA analysis

Total RNA was harvested using TRIZOL reagent (Invitrogen) followed by DNase I treatment (Roche). 1–2 µg of total RNA was reverse-transcribed using SuperScript Reverse Transcriptase III (Invitrogen) with random hexamers (Invitrogen), oligodT primer (for polyA+ RNA) or gene-specific reverse primer (Table S1). The qPCR primers for amplification of polyA+ RNAs were the following: β-actin (ex5F/ex6R), GAPDH (F/R3), γ-actin (γ-actin spliced F/R), *FXN* (ex3F/ex4R), *FMR1* (ex14 F/ex 15R). For analysis of nascent RNA in Figures 2A and 4C *FXN* primer FXN D was used, while in Figure 3C *FXN* primer B was used. For quantitative real-time PCR, 2 µl of cDNA was analyzed using a Rotor-Gene RG-3000 real-time PCR machine (Corbett Research) with QuantiTect SYBR green (Qiagen). For analysis of nascent *FXN-Luc* RNA in HEK293 cells, lucR primer was used for reverse transcription, and in4F and ex5R were used for qPCR.

### RNAi and protein analysis

The RNAi was carried out as described [38]. Control siRNA duplex was 5′-UAGCGACUAAACACAUCAA -3′ (Thermo Scientific siGENOME Non-Targeting siRNA #1D-001210-01-20), RNase H1 siRNA duplex was s48357 (Ambion). mRNA target sequence for Topoisomerase I siRNA duplex was 5′-GGACUCCAUCAGAUACUAU -3′. Total protein extracts were harvested using RIPA buffer. 20 and 40 µg of total protein extracts were resolved on SDS-PAGE and detected by Western blotting. Western blots were probed with Topoisomerase I (AbCam, ab109374), actin (Sigma, A2066), RNase H1 (AbCam, ab83179) antibodies.

### RNase H1 over-expression

*FXN-Luc* and *FXN-GAA-Luc* cells were freshly split into 10 cm dishes and transfected on the following day with 10 µg Flag or RNase H1-Flag plasmids using TransFectin reagent (BioRad), following the manufacturer's instructions. Cells were harvested 48 hours after transfection. RNaseH1-Flag plasmid was cloned by replacing the GFP tag in the RNaseH1-GFP plasmid, provided by Prof.R.J. Crouch, with the Flag tag using RNaseH1-FLAG(F) and RNaseH1-FLAG(R) primers.

### Br-UTP nuclear run-on analysis

The Br-UTP NRO analysis was carried as described in [16]. The equivalent of 8×10^6^ nuclei from lymphoblastoid cells were used for each Br-UTP NRO reaction.

### Statistical analysis

Unless otherwise stated, the figures present the average values of at least three independent experiments +/− SEM. Asterisks (*) indicate statistical significance (* p<0.05; ** p<0.01; *** p<0.001), based on unpaired, two-tailed distribution Student's t test.

## Supporting Information

Figure S1 Histone H3 ChIP on *FXN* gene. Histone H3 ChIP in control and FRDA cells. γ-actin is used as positive control. Bars are average values +/− SEM (n>3).
(PDF)

Figure S2 DIP and nascent RNA analysis in additional control (GM06895, GM14926) and FRDA (GM16209, GM16243) cells. A, B. Left panel: RT-qPCR analysis of *FXN* nascent RNA in two control (A-GM06895, B-GM14926) and two FRDA (A-GM16209, B-GM16243) cells, normalised to 5S rRNA and relative to ex1 RNA in control cells. A, B. Right panel: DIP on endogenous *FXN* gene in two control (A-GM06895, B-GM14926) and two FRDA (A-GM16209, B-GM16243) cells. γ-actin is positive control. C. DIP analysis on endogenous *FXN* gene in three control and three FRDA cells. The values are normalized to ex1 amplicon in control cells. Positions of the qPCR amplicons are on the X axis; relative DIP signal is on the Y axis. Bars in A–C are average values +/− SEM (n>3).
(PDF)

Figure S3 R-loops on expanded repeats of *FXN* gene are degraded following actinomycin treatment for 48 h. DIP on *FXN* gene in control and FRDA cells treated with 5 µg/ml of actinomycin D for 48 hours. γ-actin is positive control. Bars are average values +/− SEM (n>3).
(PDF)

Figure S4 H3K9me2 ChIP in *FXN-Luc* and *FXN-GAA-Luc* HEK293 cells. A. H3K9me2 ChIP on *FXN* gene in *FXN-Luc* and *FXN-GAA-Luc* HEK293 cells. H3K9me2 levels were normalized to the total H3 levels. B. RT-qPCR analysis of RNase H1 mRNA in *FXN-Luc* and *FXN-GAA-Luc* HEK293 cells, over-expressed with Flag and RNase H1-Flag plasmids. Values are normalised to the level of GAPDH mRNA and are relative to *FXN-Luc* cells, over-expressed with Flag. C. RT-qPCR analysis of *FXN-Luc* nascent RNA in *FXN-Luc* and *FXN-GAA-Luc* HEK293 cells, over-expressed with Flag and RNase H1-Flag plasmids, normalized to γ-actin nascent RNA. Values are relative to *FXN-Luc* cells, treated with Flag. Bars in A–C are average values +/− SEM (n>3).
(PDF)

Figure S5 Camptothecin experiments in additional control (GM06895, GM14926) and FRDA (GM16209, GM16243) cell lines. A. DIP on *FXN* gene in control (GM14926) and FRDA (GM16243) cells, treated with 10 µM camptothecin for 6 hours. B. H3K9me2 ChIP on *FXN* gene in control (GM14926) and FRDA (GM16243) cells, treated with 10 µM camptothecin for 6 hours. H3K9me2 levels were normalized to the total H3 levels. C. RT-qPCR analysis of *FXN* nascent RNA in control (GM14926) and FRDA (GM16243) cells, treated with 10 µM camptothecin for 6 hours. Values are relative to untreated control cells and normalized to γ-actin nascent RNA. D. RT-qPCR analysis of *FXN* nascent RNA in control (GM06895) and FRDA (GM16209) cells, treated with 10 µM camptothecin for 6 hours. Values are relative to untreated control cells and normalized to γ-actin nascent RNA. Bars in A–D are average values +/− SEM (n>3).
(PDF)

Figure S6 H3K9me2 ChIP on *ACTB* gene. A. Diagram of *ACTB* gene. Black boxes are exons, white boxes are 5′ and 3′UTRs, lines are introns, grey box is the pause element, essential for the process of Pol II transcriptional termination [16]. TSS is the transcriptional start site. qPCR amplicons are shown below the diagram. B. H3K9me2 ChIP in control (GM15851) and FRDA (GM15850) cells. H3K9me2 levels were normalized to the total H3 levels. γ-actin is used as background control. Bars are average values +/− SEM (n>3).
(PDF)

Table S1 Sequences of PCR primers.
(DOCX)
