# Supplementary figures and images for: R-loops Associated with Triplet Repeat Expansions Promote Gene Silencing in Friedreich Ataxia and Fragile X Syndrome
Source: PLoS Genet. 2014 May 1;10(5):e1004318. doi: 10.1371/journal.pgen.1004318 (PMC4006715; doi:10.1371/journal.pgen.1004318)

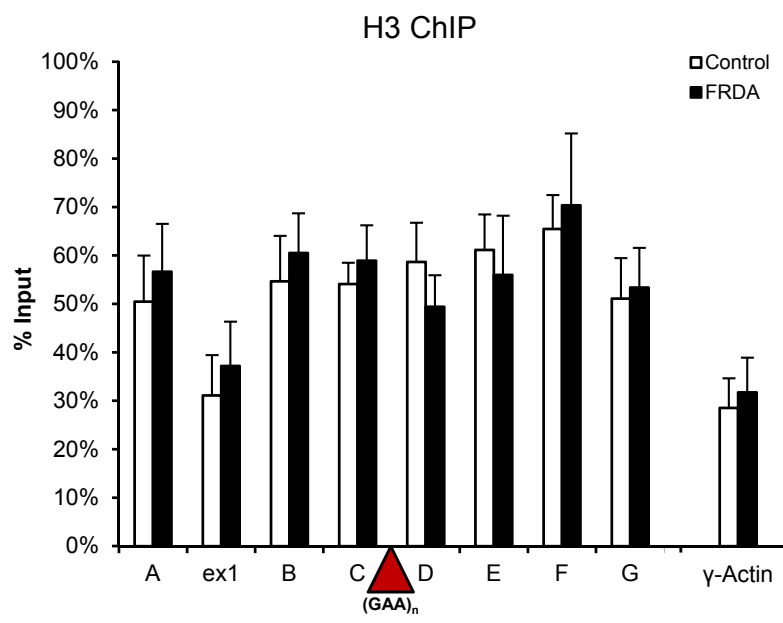

Figure S1

Supplement: Figure S1 — Histone H3 ChIP on FXN gene. Histone H3 ChIP in control and FRDA cells. γ-actin is used as positive control. Bars are average values +/− SEM (n>3). (PDF) [file pgen.1004318.s001.pdf]

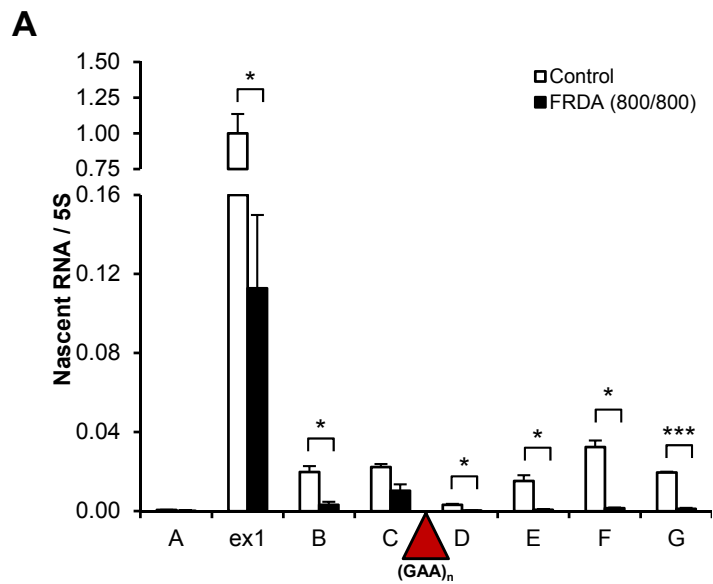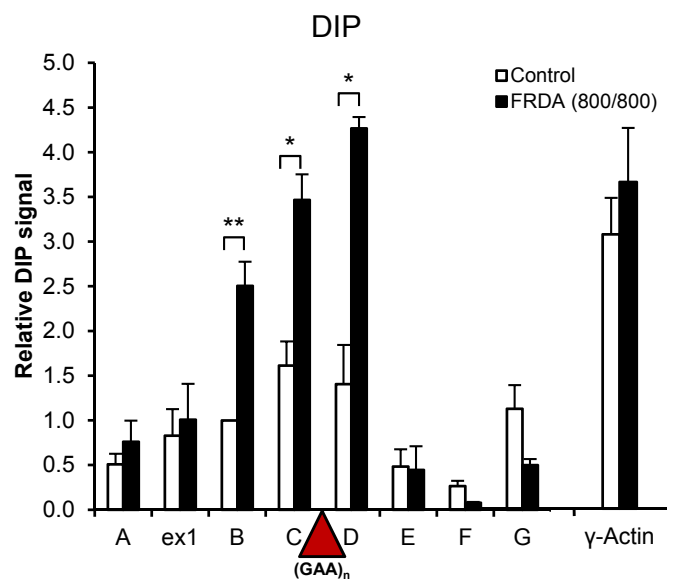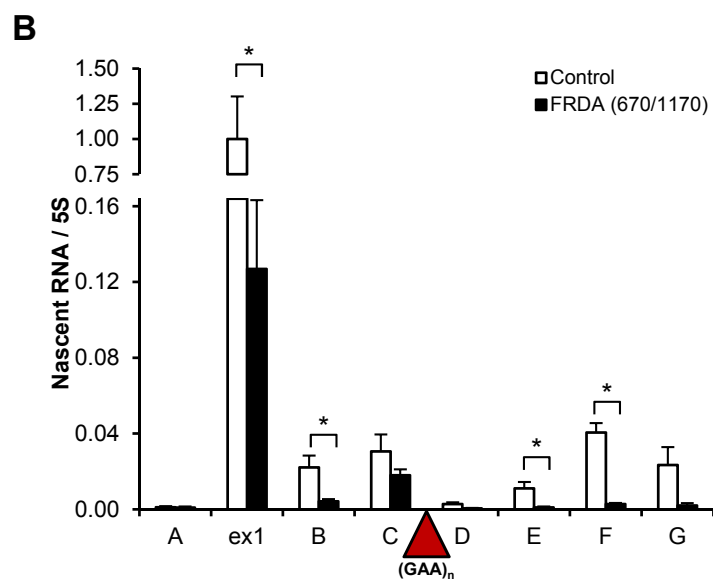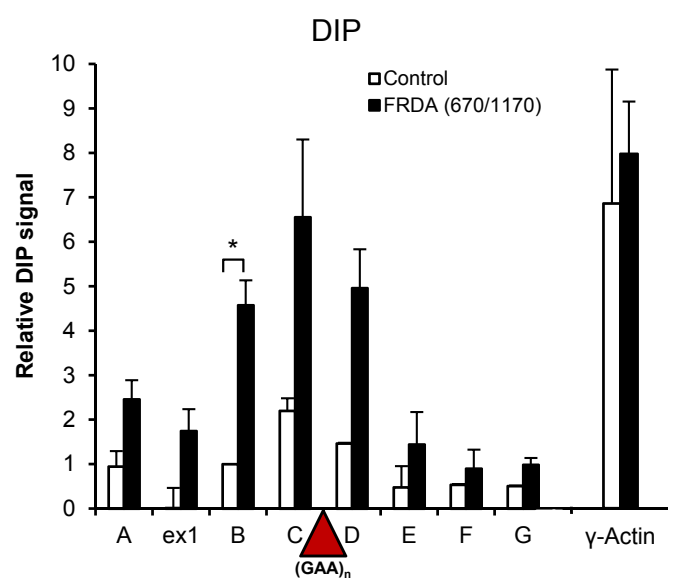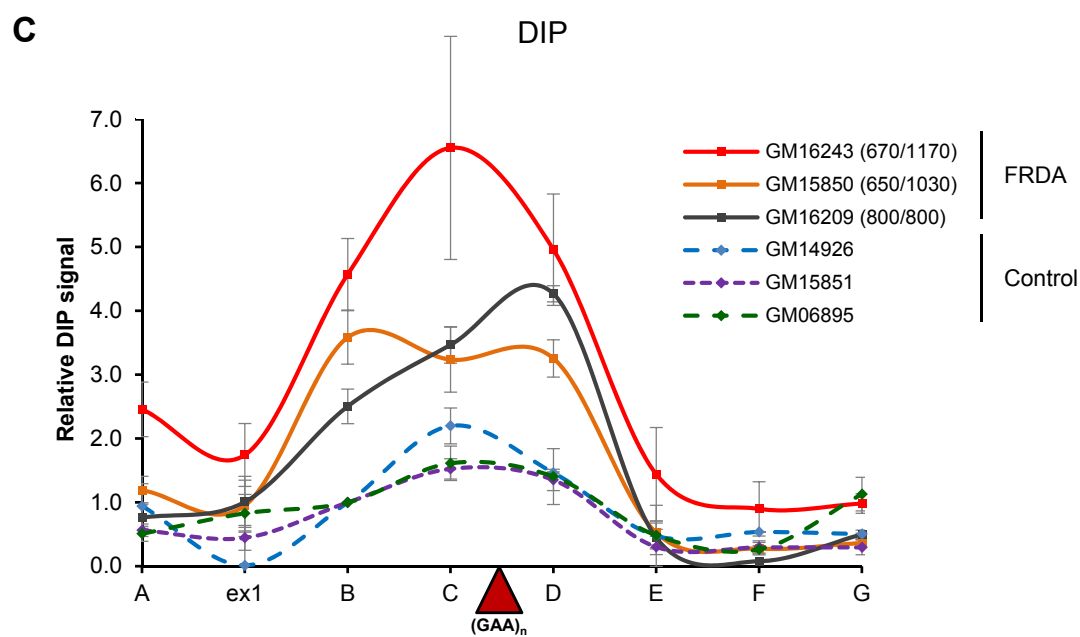

Figure S2

Supplement: Figure S2 — DIP and nascent RNA analysis in additional control (GM06895, GM14926) and FRDA (GM16209, GM16243) cells. A, B. Left panel: RT-qPCR analysis of FXN nascent RNA in two control (A-GM06895, B-GM14926) and two FRDA (A-GM16209, B-GM16243) cells, normalised to 5S rRNA and relative to ex1 RNA in control cells. A, B. Right panel: DIP on endogenous FXN gene in two control (A-GM06895, B-GM14926) and two FRDA (A-GM16209, B-GM16243) cells. γ-actin is positive control. C. DIP analysis on endogenous FXN gene in three control and three FRDA cells. The values are normalized to ex1 amplicon in control cells. Positions of the qPCR amplicons are on the X axis; relative DIP signal is on the Y axis. Bars in A–C are average values +/− SEM (n>3). (PDF) [file pgen.1004318.s002.pdf]

# DIP + 48h Actinomycin D

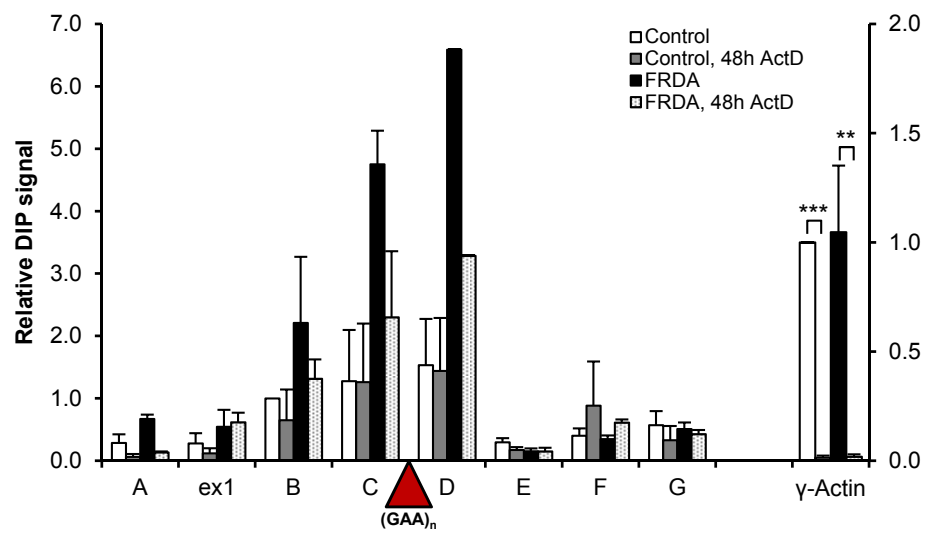

Supplement: Figure S3 — R-loops on expanded repeats of FXN gene are degraded following actinomycin treatment for 48 h. DIP on FXN gene in control and FRDA cells treated with 5 µg/ml of actinomycin D for 48 hours. γ-actin is positive control. Bars are average values +/− SEM (n>3). (PDF) [file pgen.1004318.s003.pdf]

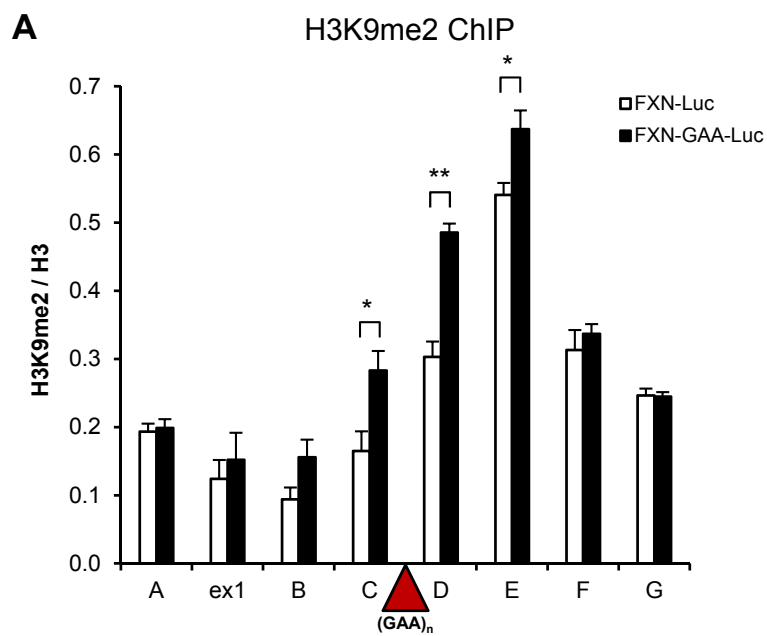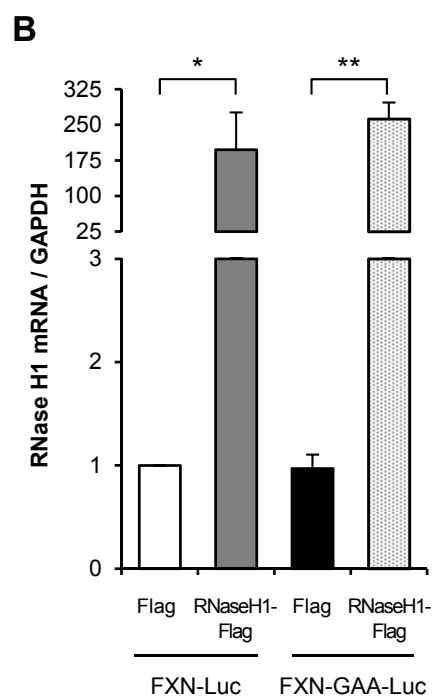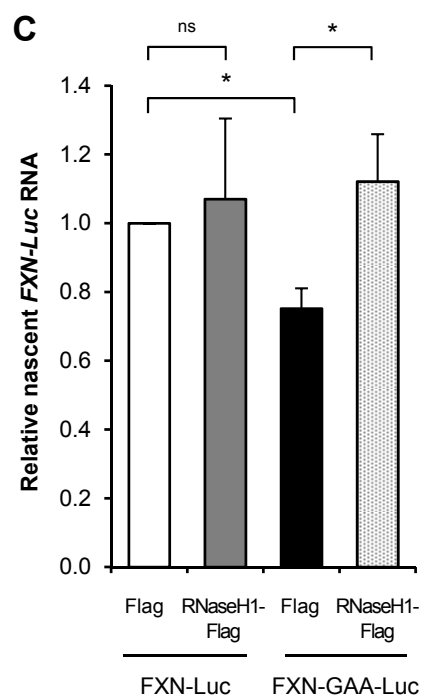

Figure S4

Supplement: Figure S4 — H3K9me2 ChIP in FXN-Luc and FXN-GAA-Luc HEK293 cells. A. H3K9me2 ChIP on FXN gene in FXN-Luc and FXN-GAA-Luc HEK293 cells. H3K9me2 levels were normalized to the total H3 levels. B. RT-qPCR analysis of RNase H1 mRNA in FXN-Luc and FXN-GAA-Luc HEK293 cells, over-expressed with Flag and RNase H1-Flag plasmids. Values are normalised to the level of GAPDH mRNA and are relative to FXN-Luc cells, over-expressed with Flag. C. RT-qPCR analysis of FXN-Luc nascent RNA in FXN-Luc and FXN-GAA-Luc HEK293 cells, over-expressed with Flag and RNase H1-Flag plasmids, normalized to γ-actin nascent RNA. Values are relative to FXN-Luc cells, treated with Flag. Bars in A–C are average values +/− SEM (n>3). (PDF) [file pgen.1004318.s004.pdf]

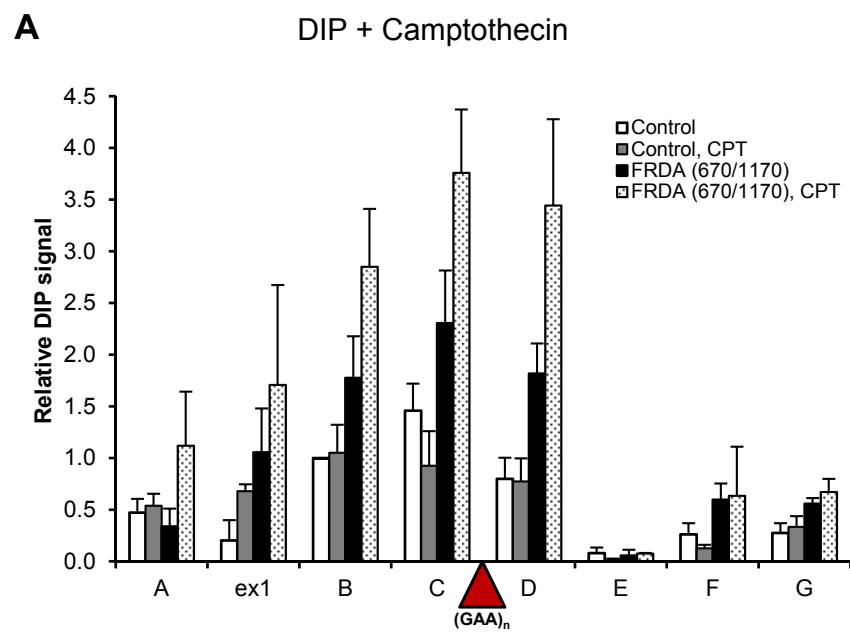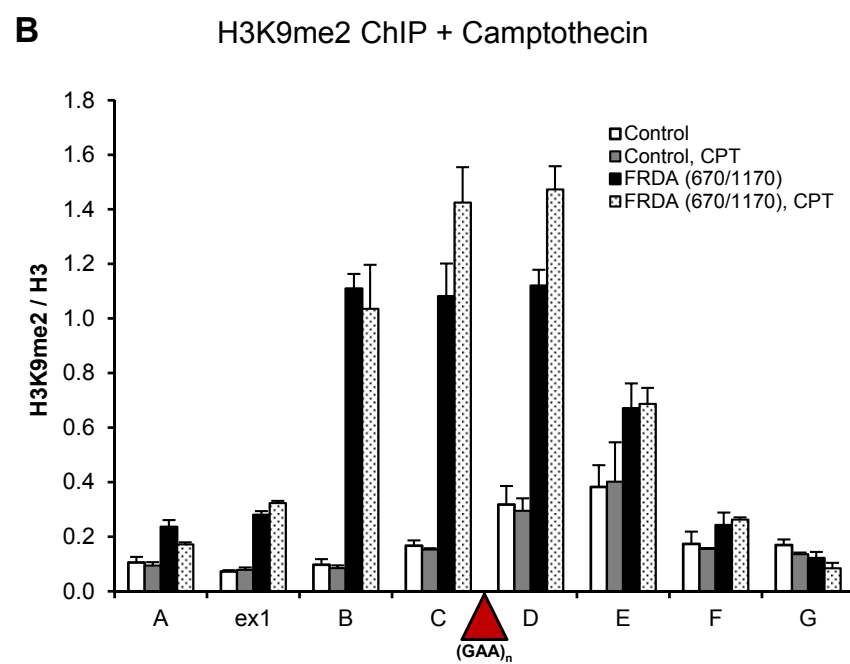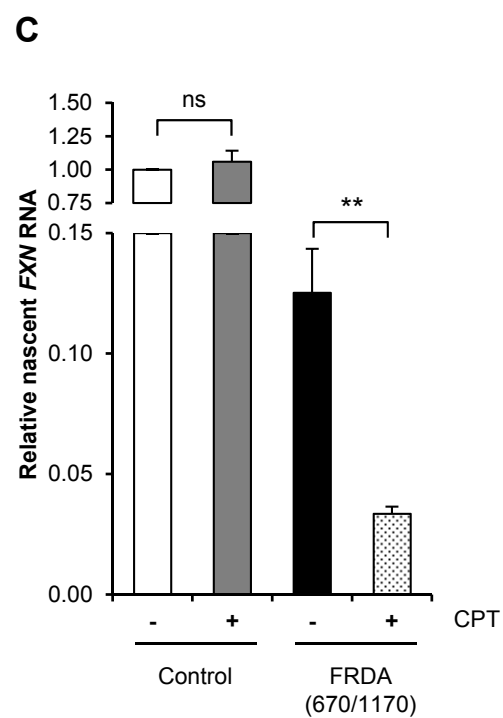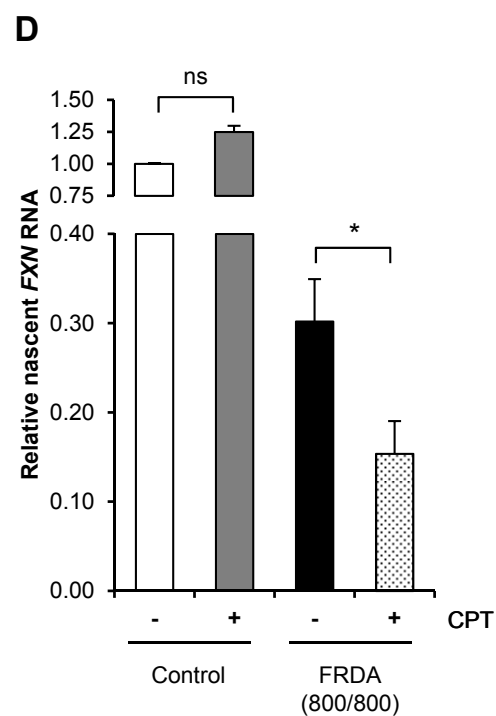

Figure S5

Supplement: Figure S5 — Camptothecin experiments in additional control (GM06895, GM14926) and FRDA (GM16209, GM16243) cell lines. A. DIP on FXN gene in control (GM14926) and FRDA (GM16243) cells, treated with 10 µM camptothecin for 6 hours. B. H3K9me2 ChIP on FXN gene in control (GM14926) and FRDA (GM16243) cells, treated with 10 µM camptothecin for 6 hours. H3K9me2 levels were normalized to the total H3 levels. C. RT-qPCR analysis of FXN nascent RNA in control (GM14926) and FRDA (GM16243) cells, treated with 10 µM camptothecin for 6 hours. Values are relative to untreated control cells and normalized to γ-actin nascent RNA. D. RT-qPCR analysis of FXN nascent RNA in control (GM06895) and FRDA (GM16209) cells, treated with 10 µM camptothecin for 6 hours. Values are relative to untreated control cells and normalized to γ-actin nascent RNA. Bars in A–D are average values +/− SEM (n>3). (PDF) [file pgen.1004318.s005.pdf]

**A**

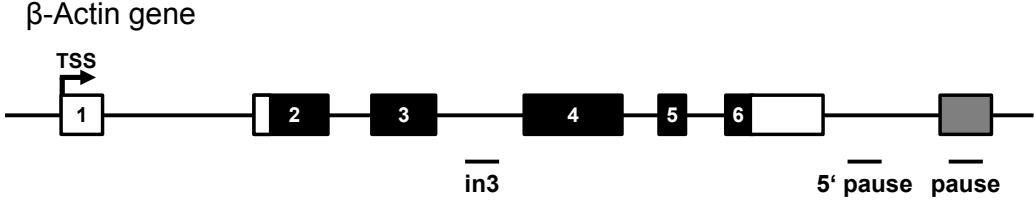

**B**

H3K9me2 ChIP

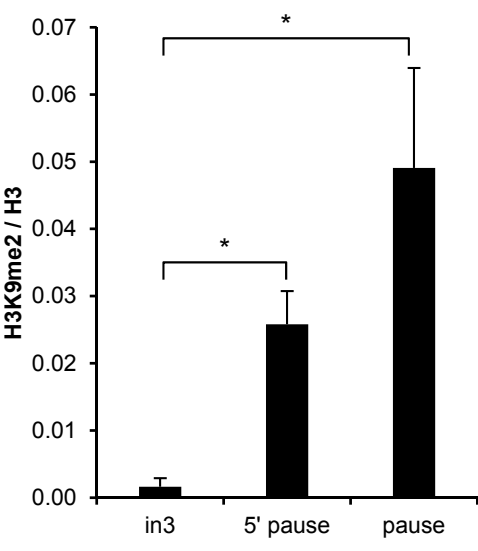

Supplement: Figure S6 — H3K9me2 ChIP on ACTB gene. A. Diagram of ACTB gene. Black boxes are exons, white boxes are 5′ and 3′UTRs, lines are introns, grey box is the pause element, essential for the process of Pol II transcriptional termination [16]. TSS is the transcriptional start site. qPCR amplicons are shown below the diagram. B. H3K9me2 ChIP in control (GM15851) and FRDA (GM15850) cells. H3K9me2 levels were normalized to the total H3 levels. γ-actin is used as background control. Bars are average values +/− SEM (n>3). (PDF) [file pgen.1004318.s006.pdf]
