# Supplementary material for: R-loops Associated with Triplet Repeat Expansions Promote Gene Silencing in Friedreich Ataxia and Fragile X Syndrome
Source: PLoS Genet. 2014 May 1;10(5):e1004318. doi: 10.1371/journal.pgen.1004318 (PMC4006715; doi:10.1371/journal.pgen.1004318)
Supplement: Table S1 — Sequences of PCR primers. (DOCX) [file pgen.1004318.s007.docx]

**Supplementary Table S1**: PCR primers

| **Name** | **Sequence (5’ 3’)** |
| --- | --- |
| ***FXN*** |  |
| A (F) | CCCCACATACCCAACTGCTG |
| A (R) | GCCCGCCGCTTCTAAAATTC |
| Ex1 (F) | GAGCAGCATGTGGACTCTCG |
| Ex1 (R) | AGAGTGGGGCCAACTCT |
| B (F) | AAACTGACCCGACCTTTATTCCA |
| B (R) | GGAATCCCCCAAGGTCACA |
| C (F) | GAAACCCAAAGAATGGCTGTG |
| C (R) | TTCCCTCCTCGTGAAACACC |
| D (F) | CTGGAAAAATAGGCAAGTGTGG |
| D (R) | CAGGGGTGGAAGCCCAATAC |
| E (F) | ACTTCAGGCCCAGAAACTATCTAATATT |
| E (R) | TGCCTAGCTTGAGGCTTCAGA |
| F (F) | TGGCTTTCAGAGTTCGAACCA |
| F (R) | CACTCTGCTTTTTGACATTCCAA |
| G (F) | TGACCAGAGTACTTTGCATGAATGT |
| G (R) | CCAAACGCAGCTGCTAGAATATT |
| UP (F) | CTGACCCGACCTTTATTCCA |
| UP (R) | TGGGCGTCACCTTTATCTTC |
|  |  |
| FXN spliced ex3 (F) | CAG AGG AAA CGC TGG ACT CT |
| FXN spliced ex 4 (R) | AGC CAG ATT TGC TTG TTT GG |
|  |  |
| ***FMR1*** |  |
| FMR1 UP (F) | ACAGTGGAATGTAAAGGGTTG |
| FMR1 up (R) | GTG TTA AGC ACT TGA GGT TCAT |
| FMR1 ex 1 (F) | GAA CAG CGT TGA TCA CGT GAC |
| FMR1 ex 1 (R) | GTG AAA CCG AAA CGG AGC TGA |
| FMR1 in1A (F) | TAAATTCAGGAATGCACATGC |
| FMR1 in1A (R) | CCT GAA GTT TCA TGG CAT ATA TT |
| FMR1 in 1B (F) | CTT GAA GGT GAA TGA AGA ATA GG |
| FMR1 in 1B (R) | AGC AAT TTG TCT GAC ACA CAC |
| FMR1 in 15 (F) | GAACTTCCAGTAAGCATTTCAG |
| FMR1 ex 16 (R) | CTG TTG TTC TTC CTT TAG CCT CTC |
| FMR1 ex 14 spliced (F) | GGA GCT AGT TCT AGA CCA CCA C |
| FMR1 ex 15 spliced (R) | GAG TTC GTC TCT GTG GTC AGA TTC |
|  |  |
| **γ-actin** |  |
| intr1 (F) | CCG CAG TGC AGA CTT CCG AG |
| intr1 (R) | CGG GCG CGT CTG TAA CAC GG |
| γ-actin spliced (F) | AAT CTT GCG GCA TCC ACG AG |
| γ-actin spliced (R) | TCG TAC TCC TGC TTG CTA ATC C |
|  |  |
| **GAPDH** |  |
| GAPDH (F) | ACA TCA AGA AGG TGG TGA AG |
| GAPDH (R3) | GGG TCT TAC TCC TTG GAG GC |
|  |  |
| **β-actin** |  |
| β-actin ex5 (F) | GGA CAT CCG CAA AGA CCT GTA |
| β-actin ex6 (R) | CTC CAA CCG ACT GCT GTC ACC |
|  |  |
| 5S (F) | AGC GTC TAC GGC CAT ACC |
| 5S (R) | GGT ATT CCC AGG CGG TCT C |
|  |  |
| **RNase H1** |  |
| RNaseH1 (F) | TAACTGGGTTCAAGGTTGGAAG |
| RNaseH1 (R) | TCTTCCGATTGTTTAGCTCCTTC |
| RNaseH1-FLAG (F) | GATTACAAGGATGACGACGATAAGGTTTAAAGCGGCCGCGACTCTAGATCA |
| RNaseH1-FLAG (R) | ATCGATCCCTCCACCGTCTTC |
|  |  |
| **FXN-Luc** |  |
| in4F | GCTGTGCTGTGGAATTACT |
| ex5R | AGGCTTTAGTGAGCTCTGCG |
| lucR | TTTATGTTTTTGGCGTCTTCC |
| GAA104F | GGCTTAAACTTCCCACACGTGTT |
| GAA629R | AGGACCATCATGGCCACACTT |
|  |  |
